# Deep supercooling enabled by surface impregnation with lipophilic substances explains the survival of overwintering buds at extreme freezing

**DOI:** 10.1111/pce.13545

**Published:** 2019-04-04

**Authors:** Gilbert Neuner, Benjamin Kreische, Dominik Kaplenig, Kristina Monitzer, Ramona Miller

**Affiliations:** ^1^ Department of Botany, Unit Functional Plant Biology University of Innsbruck Innsbruck Austria‐Europe

**Keywords:** anti‐freeze substance, extrinsic ice nucleation, freezing stress, frost survival, surface ice barrier

## Abstract

The frost survival mechanism of vegetative buds of angiosperms was suggested to be extracellular freezing causing dehydration, elevated osmotic potential to prevent freezing. However, extreme dehydration would be needed to avoid freezing at the temperatures down to −45°C encountered by many trees. Buds of Alnus alnobetula, in common with other frost hardy angiosperms, excrete a lipophilic substance, whose functional role remains unclear. Freezing of buds was studied by infrared thermography, psychrometry, and cryomicroscopy. Buds of A. alnobetula did not survive by extracellular ice tolerance but by deep supercooling, down to −45°C. An internal ice barrier prevented ice penetration from the frozen stem into the bud. Cryomicroscopy revealed a new freezing mechanism. Until now, supercooled buds lost water towards ice masses that form in the subtending stem and/or bud scales. In A. alnobetula, ice forms harmlessly inside the bud between the supercooled leaves. This would immediately trigger intracellular freezing and kill the supercooled bud in other species. In A. alnobetula, lipophilic substances (triterpenoids and flavonoid aglycones) impregnate the surface of bud leaves. These prevent extrinsic ice nucleation so allowing supercooling. This suggests a means to protect forestry and agricultural crops from extrinsic ice nucleation allowing transient supercooling during night frosts.

## Abstract 2

As a fundamental new mechanism, how an overwintering angiosperm bud survives freezing, we demonstrate for A. alnobetula that the bud supercools and ice forms harmlessly in‐between the leaves. Lethal extrinsic nucleation is prevented by impregnation with lipophilic substances.

## 1 INTRODUCTION

Overwintering vegetative buds of temperate trees have been reported to survive freezing temperatures by different frost survival strategies (Sakai & Larcher, 1987). Many buds of conifers seem to use supercooling and extra‐organ freezing (Ide, Price, Arata, & Ishikawa, 1998; Picea abies Kuprian et al., 2017; *Abies* sp. Sakai, 1978; *Picea* sp. Sakai, 1979). In contrast, buds of *Pinus* species appear to tolerate extracellular freezing (Sakai, 1978). Ice seems to form harmlessly inside the bud tissue concomitantly with the stem tissue without any supercooling (Ide et al., 1998). While midwinter freezing resistance data for vegetative buds of some angiosperm species are available (Lenz, Hoch, Vitasse, & Körner, 2013; Sakai & Larcher, 1987), the frost survival mechanism itself, however, is little understood. Nevertheless, it has been suggested to be extracellular freezing tolerance (Sakai & Larcher, 1987).

During deep supercooling of buds, water migration from the supercooled bud tissues to spaces outside, that is, the subtending stem or the bud scales, has been observed. This process of freeze dehydration and external formation of ice masses has been termed extra‐organ freezing (Ishikawa & Sakai, 1982; Ishikawa & Sakai, 1985) and has been functionally associated with the maintenance of a supercooled state (Ide et al., 1998; Sakai & Larcher, 1987). Within buds that exhibit deep supercooling and extra‐organ freezing, Sakai and Larcher (1987) differentiated two types: Type I buds become fully freeze dehydrated and are considered to be fully dehydration tolerant as they can survive the temperature of LN_2_. Type II buds are not fully freeze dehydration tolerant and may not survive freezing temperatures lower than −50°C. For classification, knowledge of the extent of freeze dehydration is necessary. The extent of freeze dehydration in deeply supercooling vegetative buds of angiosperms has hardly been assessed. Lateral buds of Acer japonicum were evidenced to belong to Type I, terminal buds to Type II by estimation of freeze dehydration from shrinkage of buds in nuclear magnetic resonance (NMR) micro images (Ishikawa, Price, Ide, & Arata, 1997). After exposure to −6°C apple buds could be shown to be freeze dehydrated by −1.2 MPa. Upon their moderate midwinter maximum freezing resistance of ~−30°C (LT_i_), apple buds were assigned to Type II (Pramsohler & Neuner, 2013). Strikingly, the extent of freeze dehydration appeared not much different to that found in P. abies where buds finally get frost damaged by intracellular freezing when they cannot retain their deeply supercooled state (Kuprian et al., 2017). However, only recently it was shown that low midwinter MPa values of P. abies bud tissues have no effect on their supercooling capacity (Kuprian, Munkler, Resnyak, & Neuner, 2018). From these findings, the functional involvement of freeze dehydration or at least a dose‐effect relationship between freeze dehydration and freezing resistance of buds is not so clear.

Maintenance of supercooling in the bud cells may—alongside structural prerequisites such as ice barriers (Kuprian et al., 2017; Kuprian, Briceno, Wagner, & Neuner, 2014) and freeze dehydration—require additional currently unknown molecular components (Ishikawa et al., 2009; Ishikawa, Ishikawa, Toyomasu, Aoki, & Price, 2015; Kishimoto et al., 2014; Wisniewski, Gusta, Fuller, & Karlson, 2009; Wisniewski, Gusta, & Neuner, 2014). During supercooling such molecular components could have stabilizing activity, ice nucleation activity, and/or antifreeze activity (Ishikawa, 2014; Ishikawa et al., 2015). An interesting feature of some very frost hardy angiosperm buds (Betulaceae: *Alnus*, *Betula*; Salicaceae: *Populus*) is the excretion of a lipophilic substance containing flavonoids (Wollenweber, 1989). In buds of Alnus alnobetula glands on young bud leaves produce a sticky excretion, which contains triterpenes and flavonoid aglycones (Wollenweber, Bouillant, Lebreton, & Egger, 1971; Wollenweber, Egger, & Schnepf, 1971). The functional role of these excretions is currently unknown, but they could be involved in freezing and frost survival of the bud. In midwinter, the bud cells of A. alnobetula can survive exposure to freezing temperatures of up to −50°C (LT_50_: Benowicz, El‐Kassaby, Guy, & Ying, 2000), which is compared with other temperate angiosperm tree species quite frost hardy. The frost survival strategy of buds of A. alnobetula is unknown. Also, if—and to which extent—freeze dehydration during exposure to freezing temperatures occurs and is involved in frost survival.

We aimed to assess (1) the freezing resistance and survival strategy of vegetative buds of A. alnobetula on a seasonal basis and (2) the overall freezing pattern and behaviour in and around vegetative buds in relation to the specific bud architecture. Additionally, (3) we determined whether and—if so—buds get freeze dehydrated by movement of water towards the stem or bud scales and to what extent. (4) Finally, the findings should be related to the potential functional role of bud excretions in bud freezing resistance.

## 2 MATERIAL AND METHODS

### 2.1 Plant material

Twigs representing the “high altitude site” were collected from a natural stand of A. alnobetula (Ehrh.) K. Koch within the Alpine Garden of the Institute of Botany of the University of Innsbruck (on a north facing slope of the summit at 1,960 m a.s.l., 47.160412°N, 11.224281°E). “Valley samples” were collected from potted plants cultivated in the Botanical Garden of the University of Innsbruck at 614 m (47.160412°N, 11.224281°E), purchased as saplings that had been grown from seeds from natural A. alnobetula stands at the treeline in Tyrol by the “Landesforstgärten Tirol.” The sample material was stored wrapped up in plastic bags lined out with moist paper towels inside of a cold room at +5°C.

Overwintering buds of A. alnobetula are approximately 8 to 12 mm long, slim, egg to cone shaped, pinnacled, hairless and gluey, and appear polished (Godet, 1987). Terminal and lateral buds do not differ in their outer appearance. Terminal vegetative buds are rare as usually the reproductive buds are terminally inserted. Not only the extent of freezing resistance (Fraxinus ornus Sakai & Larcher, 1987) but also the underlying survival mechanisms (A. japonicum Ishikawa et al., 1997) have been shown to differ widely between lateral and terminal buds. Therefore, only lateral buds were used in this study.

**Figure 1:**
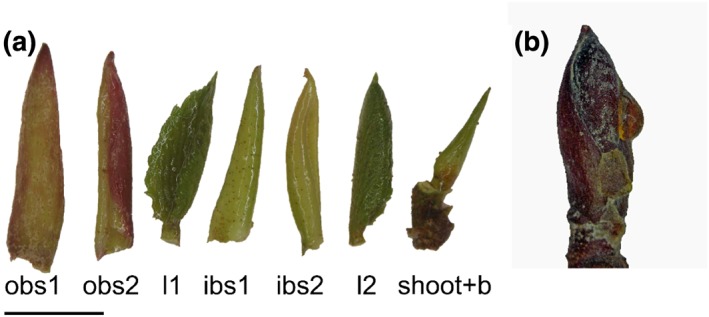
(a) Dissected overwintering bud of Alnus alnobetula collected in the Botanical Garden of the University of Innsbruck (17.10.2018). As a striking feature, all inner bud parts are thoroughly impregnated by a sticky fluid. Different bud parts are ordered from the outermost to the innermost part from left to the right: obs1 … outer bud scale1, obs2 … outer bud scale 2, l1 … basal young leaf, ibs1 … inner bud scale 1, ibs2 … inner bud scale 2, l2 … second leaf, shoot+b … bud shoot (unstrechted) and preformed bud for the year after next year bearing the shoot apical meristem. The horizontal black bar indicates 5 mm. (b) On buds of A. alnobetula, often solidified droplets of the excreted bud liquid can be found attached to the bud scales

Lateral buds of A. alnobetula contain the unstretched next year's shoot, a primary stage of the bud for the year after next year, young leaves and bud scales (Figure 1a). The typical morphology of lateral buds is as follows. Two to three green, later in the season red‐violet, bud scales enclose next year's shoot and leaf flash. Usually, two young leaves are already fairly developed and several (up to 8) mm in size. The young leaves are spaced by two inner bud scales. The scales and leaves adhere to the unstretched shoot. The shoot holds already a primary stage of the bud for the year after next year consisting of bud scales that surround the shoot apical meristem. All inner bud parts are thoroughly impregnated by a sticky fluid that is excreted by peltiform glands found on the young leaves (Wollenweber, Bouillant, et al., 1971; Wollenweber, Egger, & Schnepf, 1971). These excretions are produced to such an extent that they often leak out of the bud forming solidified droplets on the surface of bud scales (Figure 1b).

### 2.2 Freezing behavior

To assess the freezing behavior, between 15 and 20 buds were tested simultaneously during computer controlled freezing treatments, which were conducted in a laboratory freezer (Profiline Taurus 0986, National Lab, Mölln, Germany). The freezing program included a settling time of 45 min at +3°C, followed by a constant cooling rate of 4 K/h down to below the frost killing temperature of the buds. Differential thermal analysis (DTA), which allows the sensitive detection of freezing exotherms, was performed according to Burke, Gusta, Quamme, Weiser, and Li (1976) using an improved measurement procedure previously detailed in Kuprian et al. (2017). To avoid artificial supercooling the high temperature freezing exotherm, high temperature exotherm (HTE), was triggered by use of ice nucleation active (INA) bacteria (*Pseudomonas syringae* van Hall 1902). All temperatures are presented in °C and temperature differences in Kelvin (K) as is the custom in bioclimatology (Leuzinger, Vogt, & Körner, 2010).

Infrared differential thermal analysis (IDTA) allows the two dimensional measurement of freezing patterns in plant tissues and the exact localization of freezing events and ice propagation in tissues or the detection of tissues that stay free of ice, that is, supercool. Measurements were conducted with an infrared camera (FLIR S60 or FLIR T650sc, FLIR Systems, Oregon, USA) as described recently in detail by Kuprian et al. (2017); see also (Neuner & Kuprian, 2014; Wisniewski, Neuner, & Gusta, 2015). When droplets of INA bacteria suspension were used, 2 μl droplets were placed on the surface of bud leaves with a microliter syringe (Hamilton, Bonaduz, CH).

### 2.3 Freezing resistance of buds

For measurement of frost resistance, buds of A. alnobetula were exposed to controlled frost treatments that should simulate natural night frosts. Frost treatment was conducted inside of the cooling compartment of laboratory freezers that allow realizing preprogrammable temperature profiles. The laboratory freezers are permanently turned on. Depending on the actual compartment air temperature, internal heaters are turned on or off, depending on the actual set temperature of the cooling program. A fan prevents the formation of air layers and provides a homogeneous temperature distribution within the cooling compartment. After a settling time of 45 min at +3°C, the freezer was programmed to lower temperature at a rate of −3 K/h down to target temperatures. Target temperatures were held for 3 h, then samples were thawed at a rate of +5 K/h. Seven to 10 different target temperatures (at maximum 5 K difference) between +5 and −80°C were used. These target temperatures were chosen in accordance to the seasonal freezing resistance of the buds and had to be successively adjusted from summer to winter.

For frost treatment, each sampling unit consisted of 10 twigs, each of them 10 cm long and bearing three buds. The twigs were enclosed inside plastic bags that had been lined with moist paper towels. After frost treatment, these bags containing the samples were exposed at room temperature and moderate illumination (40 μmol photons.m^−2^.s^−1^) for 5 days. Control samples were stored at +5°C.

**Figure 2:**
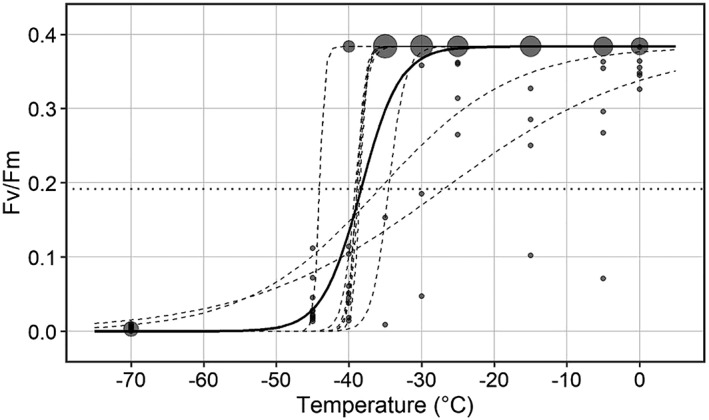
F_v_/F_m_ values as measured on dissected buds of Alnus alnobetula on 12.29.2016 8 days after a freezing test, plotted against the freezing treatment temperature. The symbol size is increased with increasing number of overlapping data points. The Boltzmann function was fitted to each of 10 data sets (hatched lines) and to all data at once (solid black line). By this, 10 LT_50_‐values were obtained at the inflection point of the sigmoidal Boltzmann curve (point of intersection with the horizontal dotted line, i.e., 50% reduction of F_v_/F_m_). Freezing resistance of buds refers to the calculated mean LT_50_ (N = 10)

For assessment of frost damage, buds were excised and longitudinally dissected with a razor blade. The cut bud surfaces were then fixed onto transparency sheets bearing 10 sample measurement spots for each exposure temperature. Fixed buds were covered with moist paper towels to prevent dehydration during subsequent dark adaption. After dark adaptation for 1 h, chlorophyll fluorescence of the buds was measured with a MINI‐PAM (WALZ, Effeltrich, Germany) using a default measuring program, which determines basic fluorescence (F_0_) followed by maximum fluorescence (F_m_) triggered by a saturating light pulse. From these parameters, F_v_/F_m_ (F_v_ being the variable fluorescence) is automatically calculated by (F_m_‐F_0_)/F_m_. F_v_/F_m_ is a measure of the maximum quantum yield of photosystem II. In untreated controls, typical PS II efficiencies (Fv/Fm) early in autumn range around 0.5. The average of F_v_/F_m_ values recorded on buds exposed to control conditions were taken to indicate 0% frost damage. For calculation of freezing resistance, values higher than this average value were set to the calculated average. Completely dead bud material (100% frost damage) effectively had an Fv/Fm of 0. Then for each of 10 data sets, F_v_/F_m_ values were plotted against the frost treatment temperature. By employment of the Boltzmann fit in R (version 3.5.1 [Feather Spray]) a sigmoidal curve was fitted to each data set (Figure 2). As a parameter of the Boltzmann function for each data set, a LT_50_ value was obtained. The LT_50_ value is the temperature at 50% damage to the sample material that equals a 50% reduction of F_v_/F_m_. Mean values of LT_50_ (*N* = 10) are shown in the figures, and LT_50_ is referred to as bud freezing resistance throughout in the text.

### 2.4 Water potential of buds

To measure the actual total water potential (Ψ_tact_) using a psychrometer, buds of A. alnobetula were isolated from the stem immediately prior to the measurements. The psychrometer consists of a control and measurement unit (PSYPRO, Water Potential System Wescor, Logan, USA) and eight separate exposure chambers (Typ C‐30‐SF Wescor, Logan, USA) enclosing the samples during determination of the water potential.

At each sampling date, Ψ_tact_ was determined for untreated bud tissues. Thereafter, in order to test whether bud tissues become freeze dehydrated during freezing, the twigs were exposed to a controlled freezing treatment in which the temperature was successively lowered at a rate of 5 K/h down to −80°C. The measurement procedure was as described in detail by Kuprian et al. (2017). It was aimed at collecting samples every 5 K, that is, −5°C, −10°C, −15°C, −20°C, −25°C a. s. o. in the sublethal freezing temperature range down to the frost killing temperature. Buds were isolated in the frozen state by detaching the bud scales and cutting of the bud tissues from the stem with a razor blade. This should ensure that water that potentially had been removed from the bud during freezing, could not be sucked up again during the process of thawing.

### 2.5 Cryomicroscopy

For cryomicroscopy, twigs of A. alnobetula were exposed to a controlled freezing treatment during the winter period 2017/2018. Cooling rate was −3 K/h down to −10°C, which was held for approximately 20 hr before the frozen buds were longitudinally dissected with a razor blade inside the cooling compartment of the freezer. The light microscope (SZX12, Olympus Austria GmbH., Vienna, Austria) was also positioned inside the freezer with oculars reaching out. Thus, the longitudinally dissected buds could be immediately placed in the frozen state on the microscope stage at −10°C and inspected for places were ice masses had grown inside the bud tissue.

## 3 RESULTS

### 3.1 Freezing resistance and risk of frost damage

**Figure 3:**
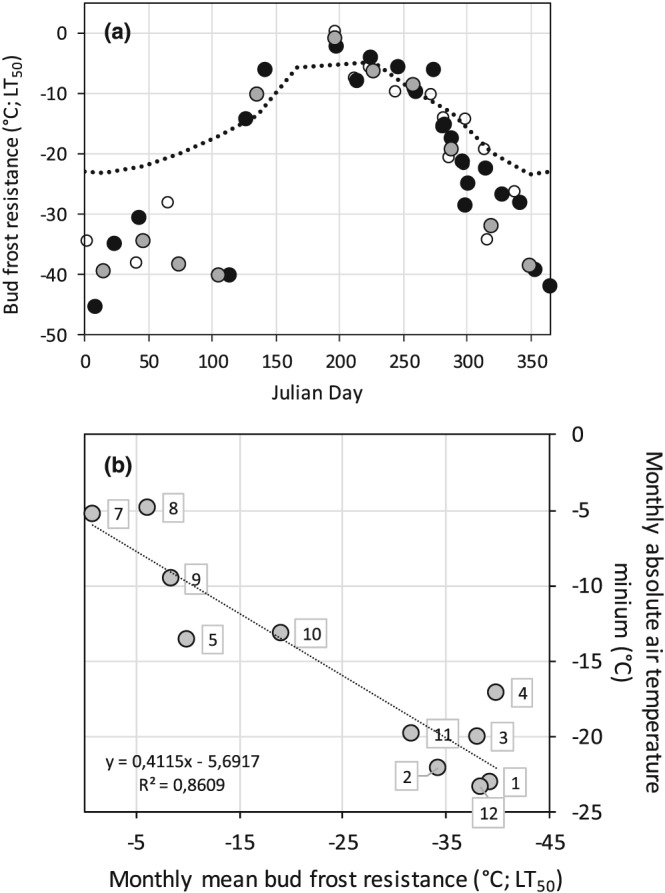
(a) Seasonal course of freezing resistance (LT_50_) of buds of Alnus alnobetula (solid circle) collected at a natural growing site (1,950 m) and (open circle) from saplings cultivated at 650 m. (grey circle) Monthly mean values of LT_50_ are additionally shown. Data were obtained between October 2013 and April 2018. The dotted line indicates the absolute air temperature minima at the high altitude site. (b) Monthly mean values of LT_50_ of the buds plotted against the absolute air temperature minima in the respective month at 1,950 m

Freezing resistance (LT_50_) of buds of A. alnobetula varied by more than 40 K with season (Figure 3). While in July, newly formed buds were very frost susceptible and could be frost damaged by −1.9°C, in midwinter they survived freezing temperatures down to −45.0°C (Figure 3a). The seasonal change of freezing resistance of buds at the high altitude and the valley site was very similar. Temperature records obtained on buds of A. alnobetula revealed that daily temperature minima of buds were not different to air temperature, but daily temperature maxima were at mean significantly warmer (+3 K; Neuner & Kreische unpublished). Within the investigation period, temperature minima of buds did not drop below −21.8°C. Even the absolute air temperature minimum at the alpine investigation site of −23.4°C is distinctly higher than midwinter freezing resistance of buds of A. alnobetula. There is a potential risk of frost damage to buds in summer (May to September) but not during winter. Monthly mean values of LT_50_ of the buds correlate with absolute air temperature minima (Figure 3b).

### 3.2 Freezing behavior and pattern

**Figure 4:**
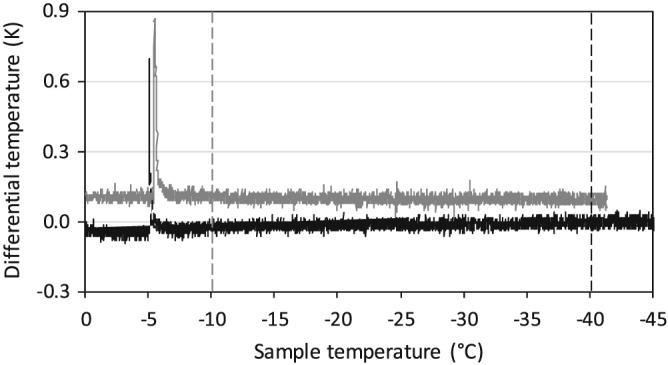
Typical differential thermal analysis (DTA) plots obtained in midwinter (black line: 1.18.2014) and at the end of summer (grey line: 9.2.2013) on buds of Alnus alnobetula during controlled freezing treatments (starting temperature +5°C, cooling rate 5 K/h, target temperature <−40°C). The freezing exotherm in the DTA plot is caused by formation of extracellular ice, that is, the so‐called high temperature exotherm, in the stem. During successively lowering of temperature down to below −40°C no further freezing event can be detected even at temperatures where the bud is frost killed (vertical hatched lines: LT_50_ grey: 9.2.2013 black: 1.18.2014)

DTA plots obtained on buds of A. alnobetula during controlled freezing as a rule showed only a single freezing exotherm (HTE; Figure 4). This freezing event was evoked by ice nucleation and freezing of apoplastic water in the stem that under the experimental conditions was artificially triggered on average at −3.4°C. Ice nucleation was triggered as otherwise, the twigs were found to remain supercooled down to −6.4°C. There is no change with season in the DTA plot. At the frost killing temperature of the buds (vertical lines LT_50_), no second freezing event could be detected by DTA.

**Figure 5:**
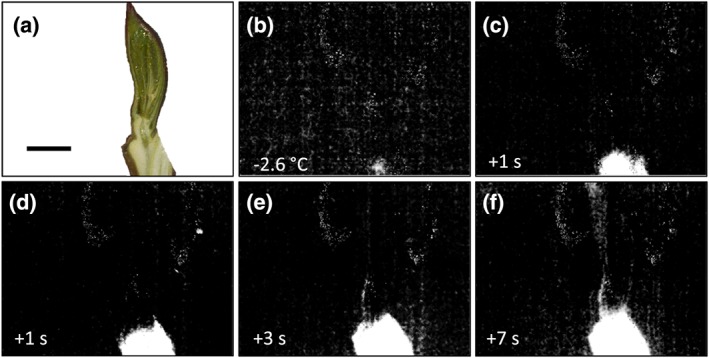
Digital colour image (a) of a longitudinally sectioned shoot bearing a vegetative bud of Alnus alnobetula before the onset of a controlled freezing treatment at 4 K/h. The horizontal bar indicates 3 mm. (b–f) Infrared differential thermal analysis images obtained during ice nucleation and high temperature exotherm (HTE) freezing of the stem. Freezing becomes visible by whitening in the images. The ice wave in the stem stopped below the bud at −2.6°C. The bud itself stayed free of ice during HTE and remained supercooled. The time span for ice propagation from ice nucleation is indicated in seconds at the bottom left corner. The whole sequence of images during stem freezing is available as video (SI. 1)

**Figure 6:**
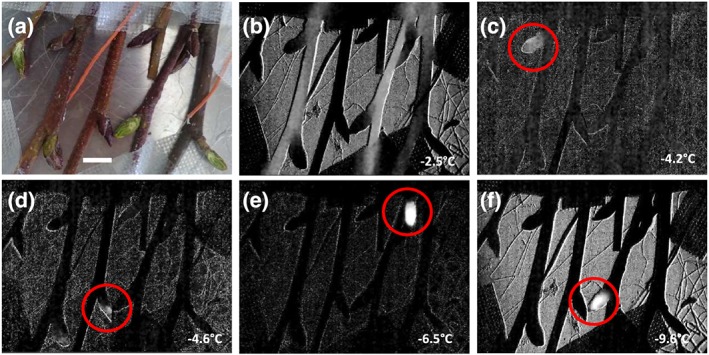
Digital colour image (a) of six twigs of Alnus alnobetula bearing intact and longitudinally sectioned vegetative buds before freezing. The horizontal white bar indicates 1 cm. (b–f) Infrared differential thermal analysis images obtained on these twigs during exposure to a controlled freezing treatment with a cooling rate of 4 K/h. Freezing becomes visible by whitening in the images. (b) During the high temperature exotherm, at −2.5°C, the stems freeze, but the buds remain unfrozen (video SI. 2). During further lowering of temperature minor freezing events originating from (c–e) intact or (f) longitudinally dissected buds (red circles) could be detected ([Link], [Link], [Link], [Link]). The temperatures when these freezing events were recorded are indicated at the bottom right corner. These freezing events were nonlethal as they occurred distinctly above the frost killing temperature of the buds (LT_50_: −20°C)

IDTA images obtained during freezing on buds corroborate the findings of DTA but additionally allow detecting tissues that supercool and places where ice masses grow. In the first IDTA example, a single bud with the adjacent stem can be seen (Figure 5; video SI. 1). After ice nucleation at −2.6°C, the ice wave spread throughout the stem but clearly stopped below the bud. This ice wave corresponds to the HTE seen in DTA. IDTA visualises that the tissues of the bud remained free of ice, that is, in the supercooled state, during this major freezing event. The second IDTA example illustrates freezing in seven twigs of A. alnobetula with some intact and some longitudinally dissected buds (Figure 6). Similarly, after ice nucleation at −2.5°C, the ice wave passed unhindered through the stems, but the buds remained unfrozen (Figure 6b; video SI. 2). Then, during further lowering of freezing temperature single, small, delimited freezing events originating from buds were visible in the IDTA images (Figure 6c–f; [Link], [Link], [Link], [Link]). These freezing events were registered at freezing temperatures distinctly above the frost killing temperature of the bud (LT_50_–20.0°C). From a single bud repeatedly during lowering of temperature such small freezing exotherms could be emitted. In no case, a freezing process around the frost killing temperature originating from the supercooled buds could be detected.

### 3.3 Cryoprotective effect of the excreted bud liquid

**Figure 7:**
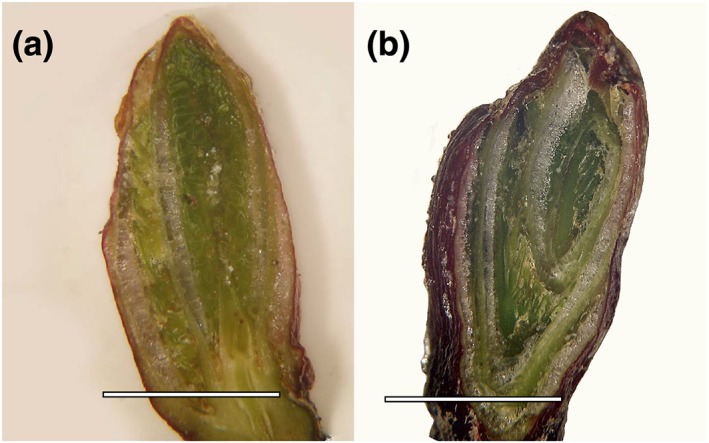
(a–b) Cross‐sectional views of buds of Alnus alnobetula. The buds had been dissected in the frozen state inside the cooling compartment of a freezer at −10°C. The image was taken with a light microscope that was also mounted inside the freezer. Ice masses can be seen between the bud scales and the young leaves inside of the bud. All bud parts remain supercooled. The horizontal white bars indicate 3 mm

**Figure 8:**
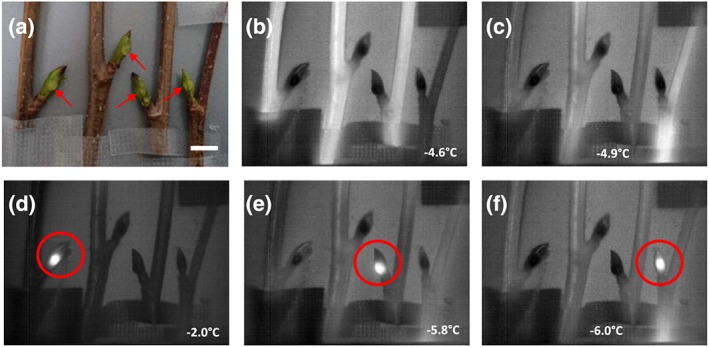
Digital colour image (a) of four twigs of Alnus alnobetula bearing vegetative buds whose outer bud scales were removed. Immediately before the onset of freezing, a 2 μl droplet of ice nucleation active (INA) bacteria suspension was deposited on the surface of the basal bud leaf of each bud (red arrows). (b–f) Infrared images were obtained on these twigs during exposure to a controlled freezing treatment with a cooling rate of 10 K/h. The horizontal white bar indicates 1 cm. Freezing becomes visible by whitening in the images. (b–c) The stems freeze during the high temperature exotherm (HTE; −4, 6/−4, 9°C), but the buds remain unfrozen (video SI. 7). Spontaneous freezing of INA droplets (d–f; red circles; [Link], [Link], [Link]) occurred independently from the HTE in the stem at deviating freezing temperatures. In no case, the INA droplets were able to nucleate the bud leaves, which remained ice free and supercooled under the experimental conditions down to −17°C

Cryomicroscopic inspection of frozen buds at −10°C revealed that ice forms inside of the buds during freezing (Figure 7a,b). These results are in accordance with the findings obtained by IDTA. Ice masses were localized between all inner bud parts, between the young leaves, the bud scales, and the bud of the year after next year. In intact buds, the same spaces are otherwise filled with the excreted bud liquid. Ice formation inside of bud scales as found in many other species was not seen. The tissues of all inner bud parts appeared to be free of ice and in a supercooled state. Artificial ice seeding by deposition of 2 μl droplets of INA bacteria suspension on bud leaves (outer bud scales were removed) was not successful to nucleate the supercooled bud leaves or other inner bud parts (Figure 8). INA bacteria droplets froze independently from the ice wave passing through the stem (Figure 8b,c) and from each other (Figure 8d–f). Frozen droplets were unable to nucleate the supercooled bud leaves, which indicates that the impregnation with the excreted bud liquid provides an extrinsic ice barrier. At −17.0°C, the samples were immediately removed from the freezer and inspected under a light microscope. The frozen droplets were not frozen to the bud leaves and could be removed. The inner bud parts appeared fully flexible and unfrozen.

### 3.4 Freeze dehydration of buds towards the stem

**Figure 9:**
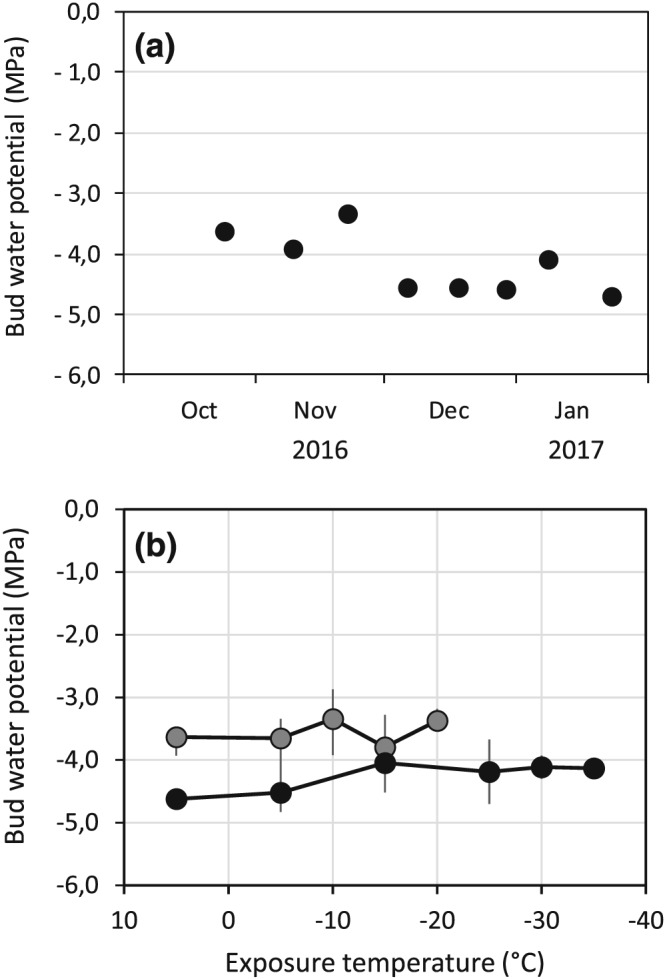
Changes of total actual water potential of bud tissues of Alnus alnobetula: (a) with season (mean values per sampling date) and (b) in response to controlled freezing treatments (starting temperature +5°C, cooling rate 5 K/h, target temperature <−40°C). Values are mean values of data obtained during freezing treatments in (grey circle) autumn (<11.24.2016, N = 3) and in (solid circle) winter (<12.6.2017, N = 5)

Throughout the season, no experimental evidence for freeze dehydration of buds of A. alnobetula towards the stem or bud scales could be obtained (Figure 9). Mean water potential (Ψ_tact_) of buds was significantly decreased from the beginning of December (Figure 9a). For determination of freeze dehydration towards subtending stem parts or outer bud scales, frozen sample twigs were removed from the freezer, and buds were immediately dissected from the stem. Also the outer bud scales were removed. These from frozen samples isolated buds had a similar Ψ_tact_ as unfrozen buds. This indicates that no water movement from the bud to the stem or bud scales occurred during freezing. Even if buds were exposed to successively lower freezing temperatures, no change in Ψ_tact_ of buds could be measured. From this, translocated ice formation in the stem or bud scales must be excluded.

## 4 DISCUSSION

Freezing resistance of vegetative angiosperm buds has scarcely been studied (Quamme, 1995; Sakai & Larcher, 1987). In midwinter buds of A. alnobetula survived freezing temperatures down to −45.0°C (LT_50_), which is much frost hardier than most of the other temperate angiosperms where bud freezing resistance is known. DTA and IDTA results suggest that the buds of A. alnobetula remain deeply supercooled down to below the frost killing temperature. This observation that the buds survive by supercooling is in contrast to the earlier suggested extracellular freezing tolerance of vegetative angiosperm buds (Sakai & Larcher, 1987). The only experimental evidence for extracellular freezing tolerance of buds exists for pines (Zwiazek, Renault, Croser, Hansen, & Beck, 2001). After the classification by Sakai and Larcher (1987), three types of supercooling buds can be differentiated. The buds of A. alnobetula are suggested to either belong to Type I or II supercooling buds. Type III can be excluded, as these buds after supercooling would show intracellular freezing upon frost damage (Kuprian et al., 2017; Kuprian et al., 2018; Rajashekar & Burke, 1978), which, using DTA and IDTA, could be demonstrated is not the case for buds of A. alnobetula. Type I buds are thought to become fully freeze dehydrated and to be fully dehydration tolerant surviving even dipping in LN_2_ when in this state (only experimental evidence: A. japonicum by NMR micro imaging; Ishikawa et al., 1997). Type II buds were suggested not to be fully freeze dehydration tolerant and become frost damaged between −35 and −50°C (apple Pramsohler & Neuner, 2013). On the basis of their supercooling behaviour and their midwinter freezing resistance (LT_50_) of −45°C, the buds of A. alnobetula can be suggested to belong to Type II.

Type II buds get freeze dehydrated, stay free of ice in the supercooled state and the removed water freezes to large extra‐organ ice masses in the subtending stem of the bud or in the bud scales (Sakai & Eiga, 1985). This process has been termed extra‐organ freezing (Ishikawa & Sakai, 1982). In supercooling buds of some species, significant freeze dehydration to the subtending stem during freezing could be measured (reproductive buds of *Rhododendron* Ishikawa & Sakai, 1982; apple Kang, Motosugi, Yonemori, & Sugiura, 1998; Pramsohler & Neuner, 2013; P. abies Type III Kuprian et al., 2017). In contrast, in excised buds of A. alnobetula, no freeze dehydration with decreasing freezing temperature could be recorded. As a striking difference to mechanisms known until now, ice masses form harmlessly between the inner bud parts of A. alnobetula. The necessary water very likely originates from freeze dehydration of the unfrozen bud parts. Therefore, when buds were excised in the frozen state at successively lower freezing temperatures, no change in water potential was observed. After the ice masses in between the young leaves of the bud melted, this water must have been reabsorbed during the water potential measurement. Still, the growth of ice masses in a certain space as seen here in A. alnobetula buds must be somehow controlled. The finding that ice was preferentially located in distinct places within plant tissues also led to the conclusion that a control mechanism for ice growth in plant tissues must exist (McCully, Canny, & Huang, 2004), whatever it may be.

Freeze dehydration of the supercooled bud has been considered to have a significant functional role as it was suggested to be involved in maintenance of the supercooled state (Ide et al., 1998). In contrast, when P. abies buds were artificially desiccated, their supercooling capacity did not increase (Kuprian et al., 2018). From this, other processes rather than freeze dehydration such as the accumulation of cryoprotective substances in cells have been suggested to be involved in the stabilization of the supercooled state (Kuprian et al., 2017). Still, buds of A. alnobetula had a deceased water potential in midwinter.

Ice formation inside the bud close to the surface of the supercooled young leaves is a new finding. Growth of ice masses between young leaves requires an efficient ice barrier against extrinsic (Pearce, 2001) ice nucleation. Otherwise, the ice on the surface would immediately cause breakdown of supercooling and lethal intracellular freezing. This was seen in buds of P. abies (Kuprian et al., 2018) that are of Type III (Kuprian et al., 2017). When spruce buds were infiltrated with water, or the gap‐less coverage by bud scales was broken by incision, the buds were unable to supercool. This led to the suggestion that the epidermal layer of the bud tissues of P. abies is no ice barrier against intrusion of surface ice. This is different in buds of A. alnobetula where ice grows harmlessly during freezing and in contact with the leaf surfaces of the young bud leaves. Therefore, the epidermal surface of the young leaves must be an effective ice barrier. This might be realised in this species by leaf surface impregnation. A peculiar feature of buds of A. alnobetula is that a hydrophobic substance is excreted during winter. The young leaves in buds of A. alnobetula possess peltiform glands that produce excretions containing triterpenes and flavonoid aglycones (Fabre‐Bonvin, Jay, & Wollenweber, 1978; Wollenweber, Bouillant, et al., 1971; Wollenweber, Egger, & Schnepf, 1971). Buds contain esters of quinic acid, ferulic acid and glycosides of quercitin, and the main sugar for glycosylation is galactose as the major flavonoid glycoside is hyperoside (Peev, Vlase, Antal, Dehelean, & Szabadai, 2007). Similar flavonoid excretions were found in other Betulaceae (*Alnus*, *Betula*, *Ostrya*) and Salicaceae (*Populus* Wollenweber, 1989), some of them having the most frost hardiest buds ever reported (−253°C Tumanov, Krasvatsev, & Khvalin, 1959). Only recently, flavonoid deposition on the surface of aerial parts of *Primula malacoides* was discussed to lower ice nucleation temperature in the leaves and thereby contributing strongly to prevent freezing damage (Isshiki, Galis, & Tanakamaru, 2014). Flavonoid glycosides (Kasuga et al., 2010; Kasuga et al., 2013) and tannin‐related polyphenols (Kuwabara et al., 2013) have also been reported to play an important role as supercooling‐facilitating (anti‐ice nucleation) active substances in xylem ray parenchyma cells of certain species. Bud excretions as found in A. alnobetula might be functionally related to the supercooling capacity of the inner bud parts. Freezing of droplets of INA bacteria suspension deposited on bud leaves were unable to nucleate the supercooled leaf. The excretions on the bud leaf surface very likely act in prevention of extrinsic ice nucleation. This allows maintaining the supercooled state despite the growth of ice masses as seen inside the buds during freezing. As their functional role was unknown till now, we propose that they have anti‐ice nucleating properties and aid supercooling of buds of A. alnobetula. Such substances with anti‐ice nucleating feature producing a barrier against extrinsic ice nucleation on plant surfaces have a high potential for application as a frost protectant in agriculture. As like, the ice growth initiated at some stage during freezing at preferential sites between inner bud parts is good evidence for specific control mechanism of preferential ice location in plant tissue.

## FUNDING INFORMATION

The experiments were conducted in the frame of research project P23681‐B16 granted by the Austrian Science Fund (FWF) to Gilbert Neuner. Ramona Miller was supported by a doctoral scholarship of the University of Innsbruck.

## CONFLICT OF INTEREST STATEMENT

There are no conflicts of interest.

## AUTHORSHIP

Gilbert Neuner designed the research and wrote the manuscript. Benjamin Kreische, Dominik Kaplenig, Kristina Monitzer, and Ramona Miller were responsible for the performance of the research, data analysis, collection, and interpretation.

## Supporting information

Video S1. Supporting information

Video S2. Supporting information

Video S3. Supporting information

Video S4. Supporting information

Video S5. Supporting information

Video S6. Supporting information

Video S7. Supporting information

Video S8. Supporting information

Video S9. Supporting information

Video S10. Supporting information
